# Experimental Study of Auxetic Structures Made of Re-Entrant (“Bow-Tie”) Cells

**DOI:** 10.3390/ma17133061

**Published:** 2024-06-21

**Authors:** Julian Plewa, Małgorzata Płońska, Kamil Feliksik, Grzegorz Junak

**Affiliations:** 1 Faculty of Science and Technology, Institute of Materials Engineering, University of Silesia in Katowice, 75 Pułku Piechoty Str., 41-500 Chorzów, Poland; malgorzata.plonska@us.edu.pl (M.P.); kamil.feliksik@us.edu.pl (K.F.); 2 Faculty of Materials Engineering, Silesian University of Technology, 8 Krasińskiego Str., 40-019 Katowice, Poland; grzegorz.junak@ps.edu.pl

**Keywords:** auxetic, re-entrant unit cell, Poisson’s ratio, arch struts

## Abstract

This article presents a study of metamaterial structures that exhibit auxetic properties. This unusual phenomenon of simultaneous orthogonal expansion of the metamaterial in tension, and vice versa in compression, with vertical and horizontal contraction, is explored for structures made of re-entrant unit cells. The geometry of such structures is analysed in detail, and the relationships are determined by the value of the Poisson’s ratio. It is shown that the Poisson’s ratio depends not only on the geometry of the unit cell but also on the degree of strain. Depending on the dimensions of the structure’s horizontal and inclined struts, the limit values are determined for the angle between them. By creating physical structures made of re-entrant cells, it is demonstrated that the mechanism of change in the structure’s dimensions is not due to the hinging but to the bending of the struts. The experimental section contains the results of compression tests of a symmetrical structure and tensile tests of a flat mesh structure. In the case of the mesh structure, a modification of the re-entrant cells was used to create arched strut joints. This modification makes it possible to obtain greater elongation of the mesh structure and larger NPR values.

## 1. Introduction

Mechanical metamaterials are a group of materials that particularly capture the researchers’ imagination. They are akin to architectural lattice structures. Their essential macroscopic property is the repeating structural units that form a continuum. They deform when subjected to a mechanical load. In the case of a tensile load, the structure expands, and conversely, in the case of a compressive load, the structure contracts. In particular, auxetic structures exhibit simultaneous contraction of the entire metamaterial in compression and its multidirectional expansion in tension. For this reason, the mechanical properties of auxetic structures are determined by negative values of the material moduli, including the Negative Poisson’s Ratio (NPR).

Producing an auxetic structure involves the creation of an array of specific repeating interconnected unit cells. To date, many different kinds of unit cells have been presented from which auxetic structures can be formed [1]. Among the best-known unit cells are the so-called ‘re-entrant honeycombs’, resembling bow-ties. The inventor of re-entrant structures is considered to be Almgren [2], who first proposed an arrangement of rigid bars connected by flexible hinges. As a result of a mechanical stimulus, a structure of this kind is deformed, with forces transmitted from one cell to an adjacent cell. Particular properties of bow-tie structures are that the material from which they are made must have elastic properties and that the arrangement of the unit cells allows them to shift, i.e., the structure must have empty spaces that can be filled as a result of deformation.

The popularity of re-entrant honeycombs is quite considerable, with an unusually large number of works being published on them, e.g., [3,4,5,6,7,8,9,10,11,12], in which attempts have been made to introduce, simulate, and analyse such structures built from re-entrant unit cells. This popular area of research and engineering interest has also been applied to the analysis of auxetic foams [13,14,15]—the first known auxetic engineering material since Lakes’ [16]. Almgren’s proposal has also seen numerous modifications and improvements to increase the stability and strength of the structures [17,18,19,20,21,22,23,24,25]. Within this group, particular attention should be paid to three-dimensional structures, e.g., [25,26,27,28,29,30], which become candidates for elements that can be subjected to large and abrupt loads.

There are also many application possibilities for bow-tie auxetic structures. They are used for designing and manufacturing auxetic materials at different scales, ranging from molecular design to engineering materials [31].

Auxetic structures are classified as so-called smart materials and have great potential in technical applications, e.g., [32], as well as in sports and medical applications [33,34]. This is due to the potentially improved mechanical properties, indentation resistance, higher shear modulus, variable permeability, and better energy absorption [34,35].

While there is a huge number of studies on structures composed of bow-ties, there is a lack of a robust engineering approach that would allow the creation of auxetic structures with specified parameters and, in particular, the construction of durable structures that do not break easily.

The work presented here attempts to fill this gap and proposes an analysis of structures made of re-entrant cells based on geometrical considerations without the commonly applied simplifications and interpolations. These considerations are backed by the physical models of structures assembled from bow-tie units, which have been mechanically tested.

## 2. Analysis of Structures Made of Re-Entrant Cells

**Figure 1:**
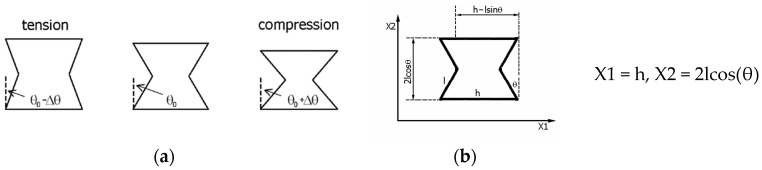
Exemplary ‘bow-tie’ re-entrant honeycomb cells—(**a**), and single ‘bow-tie’ re-entrant honeycomb cell—(**b**), where: h—horizontal strut, l—inclined strut, and θ—acute angle.

The term ‘re-entrant’ was adopted in the literature after Gibson [36,37], who first proposed lattice structures built from such unit cells in the form of a re-entrant hexagonal honeycomb system. Bow-tie unit cells referred to as ‘re-entrant’ consist of a horizontal strut h and an inclined strut l, between which there is an acute (concave) angle θ. These three parameters, h, l, and θ, are typical for ‘bow-tie’ cells (Figure 1).

Since, in auxetic structures formed from re-entrant unit cells, individual cells undergo deformation, one can study the behaviour of these cells in tension and in compression. The functioning of auxetic structures involves their movement, i.e., the change in the angle θ, in which the initial angle for the unit cells must be θ_0_, such that θ_0_ ≠ 0, and θ_0_ ≠ 90°.

It should be noted that, as a result of compression, the theta angle changes from its initial value of θ_0_ to 90°. Conversely, in tension, the angle changes from θ_0_ to 0°. It is these deformation-induced angle changes that result in a structure made up of such cells achieving an NPR. Considering an individual cell, equations for its length X1 and height X2 are provided.

Here: h—length of the horizontal strut.

l—length of the inclined strut.

θ—angle of the inclined strut.

**Figure 2:**
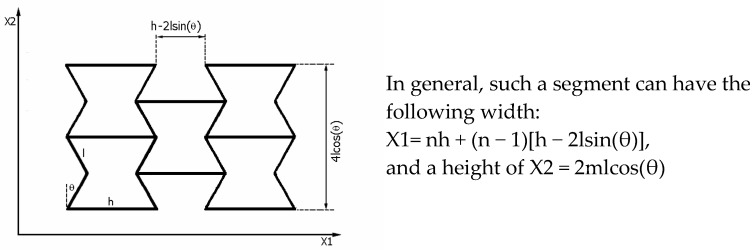
The smallest symmetrical segment of the structure and its dimensions (n = 2, m = 2); X1 = 3h-2lsin(θ) and X2 = 4lcos(θ).

In the cell structure, the inclined struts are connected to the same horizontal strut—Figure 2. It is particularly interesting that structures made of ‘bow-ties’ undergo expansion in tension, with strut joints becoming mobile. In tension, the angle θ decreases, and the structure expands. On the other hand, in compression, the angle θ increases and the structure contracts. As a result, a relative change in the linear dimensions of the structure, depending on the value of the θ angle, typically leads to an NPR.

By joining five individual cells, one obtains the simplest symmetrical segment of the structure, which, by attaching more such segments, can form a larger auxetic structure.

This is the smallest segment (five unit cells) for which a representative analysis becomes possible.

With the force applied, the structure deforms, which, depending on the direction of the force, leads to a change in the Δθ (delta theta) angle and, as a result, to expansion or contraction. Denoting the change in the dimensions by ΔX and the initial angle in the cell by θ_0_, the following expressions can be formulated to illustrate the change in the size of a structure formed horizontally from n unit cells and vertically from m columns.

The general relationships presented in Table 1 provide the basis for a theoretical analysis of structures made up of re-entrant cells.

As emphasised above, the θ angle cannot become zero in any case since that would make a re-entrant cell structure impossible, even virtually. In practice, a viable structure cannot have cells with inclined struts becoming vertical.

The above geometric relationships are sufficient to determine the Poisson’s ratio.

(1) $$\nu_{12}=-\frac{\frac{\Delta X1}{X1}}{\frac{\Delta X2}{X2}}\;\nu_{21}=-\frac{\frac{\Delta X2}{X2}}{\frac{\Delta X1}{X1}}$$

Functional relationships for the relative change in dimensions in the vertical direction, i.e., ΔX2/X2 = f(Δθ) (Table 1), yield a value between zero and one in compression. Meanwhile, in tension, the change ΔX2/X2 yields values between zero and the maximum value. It should be added that the condition θ_0_ – Δθ = 0° entails the disappearance of the re-entrant cell because it turns into a rectangle.

One more important property of the derived relationships (which are a function of the opening angle Δθ) is that they have a monotonic trend.

From a mathematical point of view, it is interesting how the value of the ratio changes ν for Δθ→0°

For Δθ = 0°, i.e., when the structure remains locked, a limit for the Poisson’s ratio value can be calculated using L’Hôpital’s rule

(2) $$\nu_{12}(\mathrm{limit})=-\frac{\cos^{2}(\theta_{0})}{\left[\frac{h}{l}-\sin(\theta_{0})]*\sin(\theta_{0}\right)}$$

This means that, with very small deformations of the structure, values of Poisson’s ratio corresponding to the limit value (2) can be reached.

**Figure 3:**
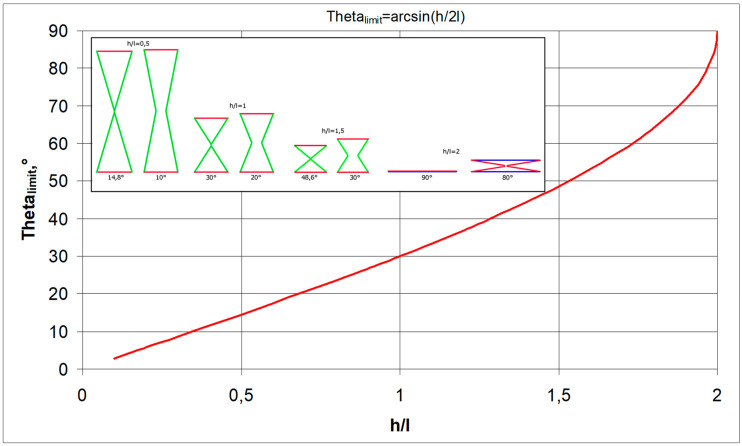
Relationship between the (h/l) ratio and the maximum value of the theta angle in compression ($\theta_{\mathrm{limit}}=\theta_{0}+\Delta \theta$) and a graphical illustration of the limit values of the angle θ for four different values of the parameter h/l.

A particular property of the presented relationships for the change in size, in the case of structures with a greater number of cells (n > 10), is that n has no effect on the value of the Poisson’s ratio. It is particularly vital to emphasise here how far the theta angle can change in tension and compression. For tension, the theta angle θ varies from θ_0_ up to 0°. For compression, on the other hand, this change is limited by the size of the struts, but for h/l > 2, the change in Δθ can be from θ_0_ to 90°. Furthermore, if h/l < 2, the inclined struts can block each other in compression (Figure 3), which corresponds to the following condition:

(3) $$\frac{h}{2l}=\sin(\theta_{\mathrm{limit}})$$

where $\theta_{\mathrm{limit}}=\theta_{0}+\Delta \theta$ is the maximum value of the angle when the structure is compressed.

The presented relationship (the geometrical constraint) indicates that for a re-entrant cell in compression, e.g., with an h/l ratio of one, the maximum value of the θ angle can be 30°. However, for h/l= 1.5, the angle θ can theoretically reach a value of 48.6°. This means in this case (h/l = 1.5 and θ_0_ = 30°) that in compression, the maximum angle θ will change by Δθ = 18.6°.

It also follows from this relationship that, for example, re-entrant cells with a parameter h/l = 1 can be made if the angle between the struts is θ_0_ < 30°. In contrast, for a re-entrant cell with the parameter h/l = 0.5, the angle θ_0_ can, at most, reach a value of 14.8°.

Figure 3 above shows the maximum values of the θ_0_ angle for the given parameter h/l, as well as the blocking condition of the struts when the cell is compressed.

### 2.1. Tension and Compression of the Structure Built of Re-Entrant Cells

**Figure 4:**
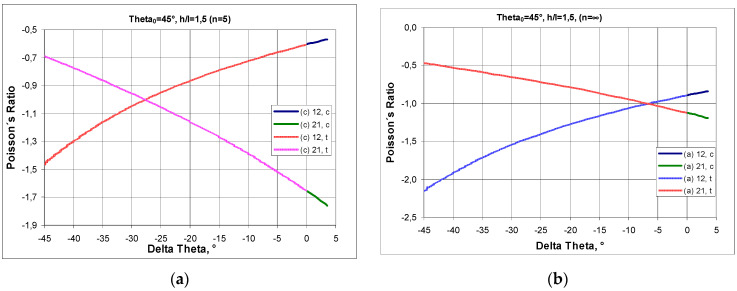
Poisson’s ratio ν_12_ and ν as a function of the change in angle Δθ (delta theta) in compression and in tension of a structure for n = 5 (**a**) and for n = ∞ (**b**), where subscript c. denotes compression and subscript t. denotes tension.

The relationships shown above for structures made up of n and m numbers of cells allow one to graphically represent the relationship between the Poisson’s ratio and the change in the angle Δθ. A structure formed from cells with a strut length ratio of h/l = 1.5 was chosen for the analysis. In general, the value of the Poisson’s ratio depends on whether the structure is in compression or in tension. The Poisson’s ratio ν_12_ increases in compression—for the change in Δθ > 0°—and decreases in tension, i.e., for Δθ < 0° (Figure 4a). An inverse trend occurs for the Poisson’s ratio ν_21_ in the transition from tension to compression, yielding higher NPR values. In compression, for h/l = 1.5, the maximum value of the angle θ is θ = 48.6°, i.e., a structure with an initial angle θ_0_ = 45° can only be compressed by Δθ = 3.6°. It is also notable that, in this case, the NPR curves for the structure in tension intersect at a particular point. For n = ∞, this intersection occurs for ν_12_ = ν_21_ = −1, with a change in the angle of Δθ= −6.5°, while in structures with a smaller number of cells (e.g., n = 5), the intersection point of the NPR curves shifts towards the lower values of Δθ (Figure 4a,b).

The accompanying graphs indicate that the analysed structure fully exhibits auxetic properties. In this case (h/l = 1.5 and θ_0_ = 45°), the Poisson’s ratio reaches moderate negative values, between −2 and −0.5.

The relative change in the linear dimensions of ΔX1/X1 also shows a characteristic pattern with a change in theta—the inclination angle.

**Figure 5:**
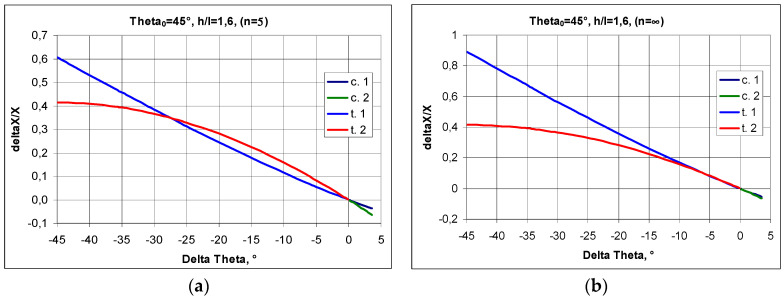
Relative change in the linear dimensions of a structure as a function of change in theta angle in compression and in tension, where subscript c. denotes compression and subscript t. denotes tension, (**a**)—for n = 5 and (**b**)—for n = ∞.

When n = 5, the same relative horizontal and vertical expansion is obtained in tension for Δθ = −27.5° (Figure 5a), which corresponds to a Poisson’s ratio value of −1.

For a structure with n = ∞, an analogous situation occurs for Δθ = −6.5°, also resulting in ν = −1.

This indicates that for small values of n, i.e., for a small number of unit cells, the value of n affects the value of the Poisson’s ratio, while for large extended structures (n→∞), this effect may not be noticeable. This is due to the simplification of the expression for ΔX1/X1 to the following form:

(4) $$\frac{\Delta X1}{X1}=\frac{-\sin(\theta_{0}+\Delta \theta )\pm \sin(\theta_{0})\;}{\frac{h}{l}-\sin(\theta_{0})}$$

When a structure with h/l < 2 is in tension, there is a large change in angle Δθ (delta theta), while in compression, the change is small—Figure 5—because the struts become blocked.

The above relationships show that by the cell count of the structure, different Poisson’s ratio values are obtained as the delta theta opening angle changes.

It should also be noted that the re-entrant cells and their joints in the structure cannot be rigid elements because they would block each other, and deformation would not be possible. Thus, the expression concerns a structure in which the cell elements (struts) can change their position, mainly due to the movable joints between them. At the very least, with the applied forces, there can be a change in the angles between the struts or their shape and length.

### 2.2. Theoretical Poisson’s Ratio Values for Auxetic Structures Built from Re-Entrant Cells

It can be seen from the above equations that the structures may function with specific values of the θ angle as well as for specific values of the h/l ratio. It is sufficient here to meet the geometrical condition, which, on the one hand, allows the structure to actually function and, on the other hand, guarantees obtaining the NPR.

Practical solutions usually use re-entrant cells for which 1< h/l < 2 occurs. In compression, for h/l = 1.5 the maximum θ angle is θ = 48.6°, while for h/l = 1.7 it is θ = 58.2°, and for h/l = 1.9 it is even θ = 71.8° (where θ = θ_0_ + Δθ), which corresponds to the blocking of the struts.

**Figure 6:**
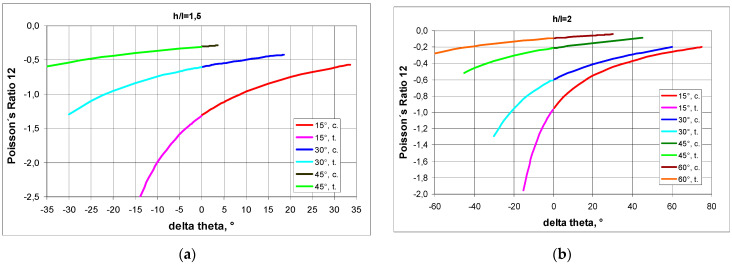
Change in Poisson’s ratio in tension (t) and in compression (c) for structures made of unit cells with a given initial value of the θ_0_ angle (assuming that Δθ is negative for tension and positive for compression), (**a**) for h/l = 1.5 and (**b**) for h/l = 2.

Presented above Figure 6 is the change in Poisson’s ratio for a structure made of re-entrant cells with a given initial value of θ_0_ and for two h/l parameters for structures in compression (c) and in tension (t).

It should be noted that if the structure is made of bow-tie cells defined by the parameter h/l = 1.5 and the angle θ_0_ = 15° then, as a result of compression, an angle change of 33.6° can occur, and the Poisson’s ratio will change from approx. −1.3 to approx. −0.56. On the other hand, as a result of tension, an angle change of 15° may occur in such a structure, and the value of the Poisson’s ratio will change from approx. −1.3 to approx. −2.6. Thus, for the structures under consideration, there is a general tendency for the Poisson’s ratio to decrease in compression and increase in tension.

The NPR decreased with the increase in the angle θ_0_ between the inclined strut and the line of the horizontal strut, both in compression and in tension.

There is a second important trend to be observed here, namely the increase in the Poisson’s ratio for small initial values of θ_0_.

Consider, for instance, a structure made of re-entrant cells with an angle of θ_0_ = 10°.

**Figure 7:**
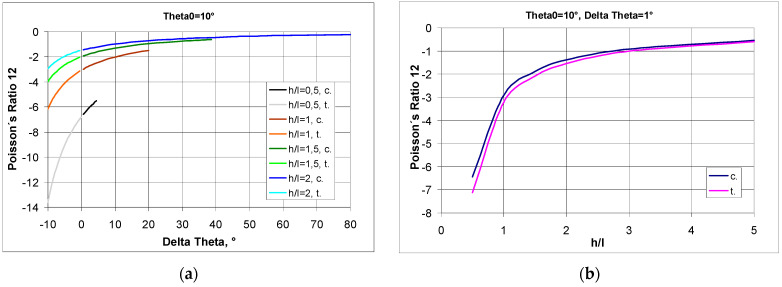
Poisson’s ratio as a function of the angle change Δθ (delta theta) for a structure with initial angle θ_0_ = 10° and for different values of the parameter h/l and the relationship between Poisson’s ratio and parameter h/l for angle change Δθ = 1° (assuming that Δθ is negative for tension and positive for compression).

In general, we find that, for h/l > 1, the Poisson’s ratio ν_12_—for compression in the X1 direction—does not vary significantly, while for compression in the X2 direction, ν_21_ varies significantly, as shown in Figure 7.

The above relationship indicates that high Poisson’s ratio values are obtained for low values of h/l (Figure 7a) and for small values of angle change (Figure 7b). With compression, the Poisson’s ratio becomes larger with an increasing angle change Δθ.

It can be concluded that as the h/l ratio increases, the Poisson’s ratio decreases, and vice versa—when the h/l ratio decreases, the Poisson’s ratio increases. This trend is particularly apparent for low values of h/l—Figure 7b. The exception is that, for such low values of h/l, the change in dimensions in the horizontal direction is much greater than the change in dimensions in the vertical direction.

**Figure 8:**
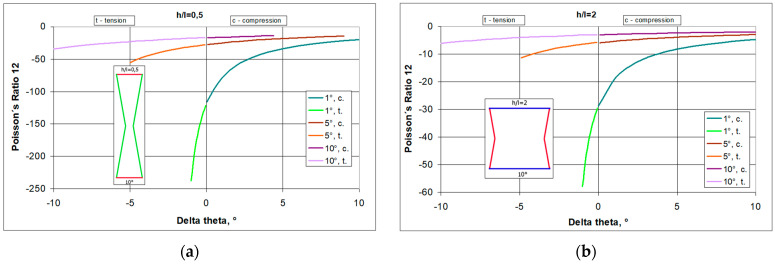
Poisson’s ratio as a function of angle change Δθ (delta theta) for structures with three values, θ_0_ = 1, 5, and 10°, and for h/l = 0.5 (**a**) and h/l = 2 (**b**) (assuming that for tension Δθ is negative and positive for compression).

The relationships shown in Figure 8 are qualitatively similar, albeit they involve different ranges of Poisson’s ratio values. They demonstrate that the smaller the initial angle θ_0_ of the re-entrant cell, the larger the Poisson’s ratio obtained. This also means that a change in the dimensions of the re-entrant cell in the horizontal direction (less elongation) has a much greater effect than an analogous change in the vertical direction.

The structure, made up of narrow bow-ties (h/l = 0.5) and with a small initial angle θ_0_, achieves very high NPR values when in tension.

### 2.3. Relationships for Small Δθ Values

Physical auxetic structures built from bow-ties yield very small changes in the angle Δθ. Thus, their change in linear size is also very small. Theoretically, this problem has been known for a long time and is described by Equation (5), which applies to very small angle changes, i.e., Δθ→0° [3]

(5) $$\nu_{12}=\frac{\cos^{2}(\theta )}{\sin(\theta )*[\frac{h}{l}-\sin(\theta )]}$$

The equation for the Poisson’s ratio limits for given initial values of θ_0_ of the bow-tie is often utilised, and it stems from Equation (2), assuming that θ_0_ = θ°. However, it is not suitable for describing any structure that undergoes very small changes in the angle Δθ. The deviations in the Poisson’s ratio values obtained from Equation (5) and from the exact Equation (1) are shown below.

**Figure 9:**
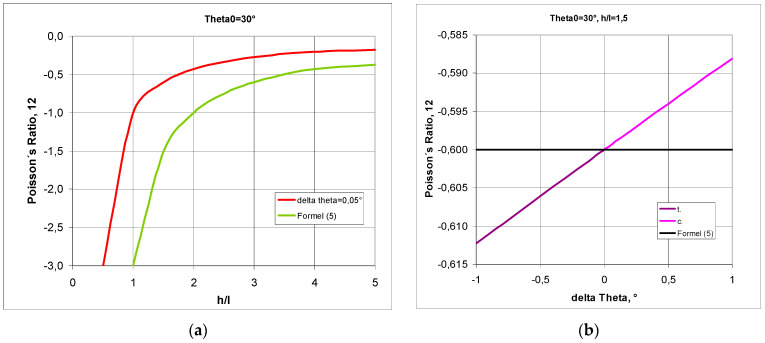
Comparison of the Poisson’s ratio curves as a function of the h/l ratio for changes in Δθ = 0.05°, and Poisson’s ratio as a function of changes in Δθ (delta theta) for h/l = 1.5, obtained from the exact and the approximate Equation (5).

The graphical relationships below are for very small changes in the delta theta angle Δθ, which also indicates small changes in the size of the structure. Using Equation (5) yields approximate values, but lower than those given by the exact Equation (1)—Figure 9a. Apart from this, the relationship obtained from Equation (5) is independent of the change in theta angle accompanying the deformation of the structure (Figure 9b).

In the theoretical considerations presented here, changes in the dimensions of the structure occur as a result of the hinge mechanism, mainly due to the coordinated movement of the inclined struts. The term hinge used in the literature to describe this process refers to the free rotation of the struts at their contact points. Since in physical structures, there are no pivot axes but rigid–flexible strut joints, other mechanisms leading to changes in the structure dimensions are mentioned, namely bending at the strut joints, as well as the bending of the struts themselves, and their tension and compression, which in macroscopic terms means the collective movement of the bow-tie elements in the structure. In general, the changes in the structure’s dimensions are not only the result of changes in the theta angle but can also be due to changes in the shape and dimensions of the struts.

At this point, it becomes clear that theoretical analysis of changes in a given structure’s dimensions is insufficient to predict the behaviour of the structure in real conditions.

A number of physical models of auxetic structures were made to consider this problem.

The theoretical analysis presented here relates to structures in which all strut joints are movable, forming a pure hinge, i.e., the shifts in the position are not constrained in any way except for the geometric constraint mentioned above. In tension and in compression, the horizontal struts move without changing their inclination angle, while the inclined struts move and rotate, thus changing their angle.

## 3. Physical Models of Structures Assembled from Struts

In the first stage of the research, the focus was put on structures made up of individual elements comprising re-entrant cells connected by hinges. These structures were acted upon in such a way that led to a change in their dimensions.

**Figure 10:**
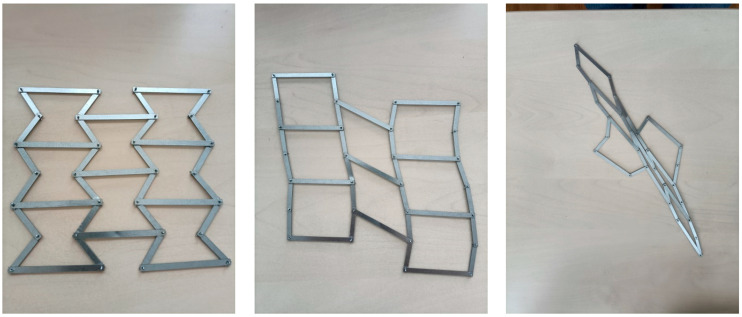
Structures made of struts connected by pivot axes in different positions.

When put in a proper position, such structures correspond to Almgren’s original concept, but through hinging, they assume an arbitrary shape and do not exhibit auxetic properties (Figure 10).

**Figure 11:**
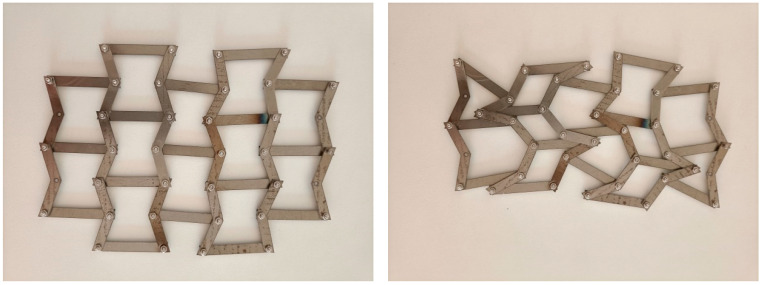
Structures made up of composite inclined struts connected to horizontal struts by pivot axes in different positions.

The following Figure 11 presents an attempt to build a structure with two types of struts connected by hinges: compound struts—made up of inclined struts—and straight horizontal struts.

The hinge mechanism itself also does not allow for uniform strain. These models demonstrate that the hinging of simple struts does not produce auxetic structures.

Thus, it can be concluded that the auxetic structure made up of re-entrant cells (bow-ties) is a dense continuum in which the essential mechanism does not involve the hinging itself but the elastic bending of the rigid–flexible strut joints (ribs).

Depending on the magnitude of the load, these ribs may also buckle.

Only by assembling the structure from elements that have rigid–flexible joints can the desired auxetic structure be obtained. In this case, structures made of re-entrant cells (bow-ties) connected by horizontal struts subjected to compressive or tensile loads as a result of their mutual interaction at the joints may be considered auxetic.

Flexible bending of strut joints can occur up to the yield strength of the material. In this case, higher degrees of expansion or contraction are achieved, but the system can no longer return (also with force applied) to its initial state (defined by θ_0_).

Physical auxetic structures containing bow-tie units were created by specially bent components or by 3D printing [38] The method used is similar to the well-known *interlocking assembly process* [39].

The first group of structures was made of PVC in a compressible form. Such a symmetrical structure does not exhibit buckling and can be subjected to cyclic compression within the elastic range. The second group of structures was in the form of meshes, which were tested in tension. In this case, the re-entrant cell structure was modified by introducing arched joints between the struts.

## 4. Symmetrical Cell Structure

**Figure 12:**
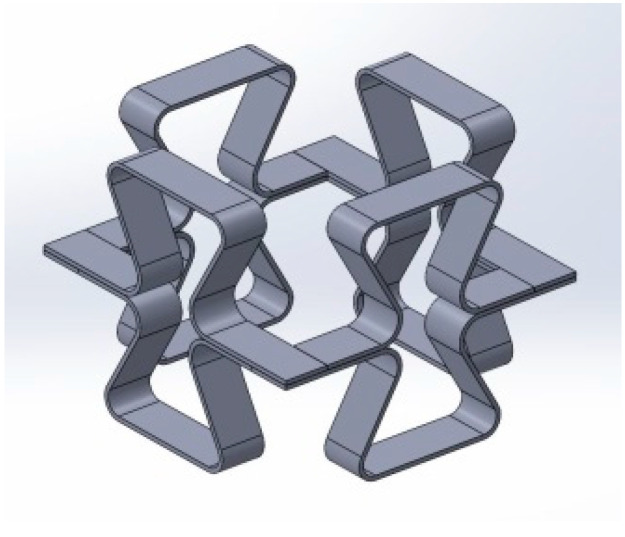
Schematic illustration of the structure created by combining four re-entrant cells.

Based on the fact that re-entrant structures buckle easily under compression, a three-dimensional structure made of four unit cells connected to each other was proposed—Figure 12.

A structure of this type is very sensitive to possible inaccuracies in the dimensions and requires very careful manufacturing, especially the precise adhesion of the horizontal struts. The structures were made from PVC strips (20 mm wide and 2 mm thick) bent hot and glued to form a three-dimensional structure, with bow-tie element parameters of h/l = 1.433 and θ_0_ = 20°.

**Figure 13:**
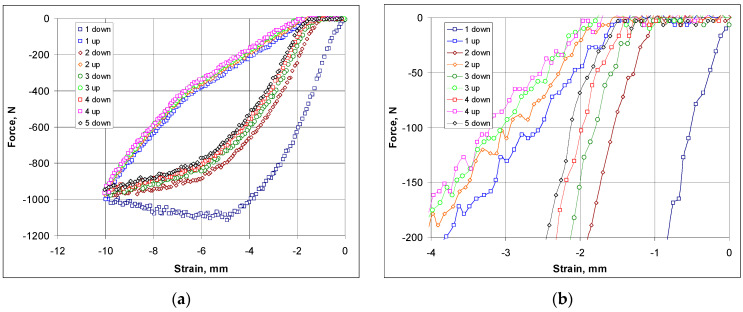
Hysteresis featuring the compression curves of the structure: applied force as a function of the achieved contraction (**a**) and the topmost part of the hysteresis (**b**).

During the compression measurement, it was found that the compression of the structure from the initial state to state X (locking of the inclined struts—the ‘contact point’) corresponding to an angle of 41° occurred in the elastic range, as shown by the hysteresis presented in Figure 13a—corresponding to cyclic compression and release.

The stress–strain curves obtained from the compression tests are shown in Figure 13a,b.

For the elastic range—Figure 13a—the curves show a repeatability of the compression and release cycle (with the exception of the curve for the first compression trial). This elastic range of the material also includes some small proportion of the plastic range—as indicated precisely by the hysteresis slice shown in Figure 13b. A slight shortening of the structure is observed after each compression–release cycle.

It has also been found that, in the elastic range, all four connected unit cells deformed evenly.

**Figure 14:**
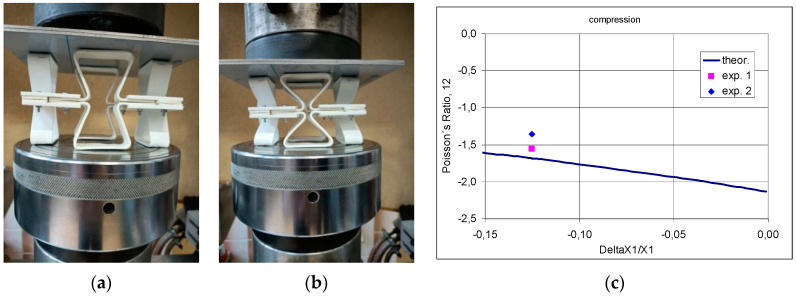
Photograph of the structure under compression in two positions: initial position (**a**) and end position (X position) (**b**) and the corresponding Poisson’s ratio relationship (**c**).

The determined experimental value of the Poisson’s ratio corresponds to the transition between the positions—Figure 14a,b. The Poisson’s ratio values resulting from changes in the linear dimensions of the structure testify to its auxetic behaviour and approach the calculated theoretical values (Figure 14c). Theoretical considerations show that the Poisson’s ratio depends on the level of strain.

When the structure is subjected to compression with larger forces, the structure collapses, which corresponds to a typical linear stress–strain curve, including the elastic range already shown and the plastic range with its characteristic plateau.

The change in the linear dimensions in the maximally compressed and broken structure affected the Poisson’s ratio. During the process of breaking the structure, the Poisson’s ratio assumed positive values.

**Figure 15:**
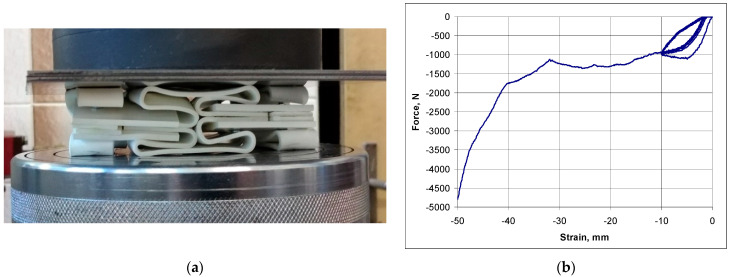
Photograph of a maximally compressed structure (**a**) and the stress–strain curve (**b**).

Once the plateau was passed (Figure 15b), a compaction of the structure occurred, with its elements coming into contact and the failure load exceeding the force values while at the plateau by a factor of three.

It is known from theory that the stress–strain curve indicates not only the effective stiffness and strength of the cell material but also the total amount of absorbed energy, which is equal to the area under the curve. Its value was estimated at around 480 J.

In the experimental compression test in the elastic range, a strain value of 12.5% was obtained in the vertical direction, while the final strain in the plastic range—at the structure’s breaking point (Figure 15a)—was 62.5%.

### Discussion of Symmetrical Cell Structure

The design of an auxetic structure made up of four connected re-entrant cells forming a closed system may resemble an automotive suspension system. Similar three-dimensional structures based on bow-tie elements have already been described in a number of research papers [26,40,41,42,43,44,45,46]. The combined individual cells, forming a two-dimensional structure, can easily be transformed into a three-dimensional structure that exhibits auxetic behaviour.

This type of structure is particularly useful for demonstrating auxetic properties in compression.

The dynamic behaviour of the three-dimensional structure is characterised by a significant increase in the level of compressive force in the various stages of compression. The observed rapid compaction at the final stage of compression appears to be a typical phenomenon [42]

Such structures can be considered useful in terms of their ability to absorb abrupt mechanical impact, as shown in earlier studies [26,40,41,42,43,44,45,46]. In practice, protective elements that break under such abrupt impact should also be economical. Thus, it is conceivable that a low-cost polymeric material (e.g., PVC) could be a candidate for such applications.

For the elastic range, the structure under study can act as a shock absorber, as shown in earlier work [40]. Although the tested structure is made up of only four unit cells, it exhibits very high structural and mechanical stability. On the other hand, the structure appears complicated to produce, but thanks to the assembly method used, it is easy to manufacture and can fulfil most functional applications.

## 5. Mesh Structures

The study utilised a specially designed re-entrant structure with arched strut joints. Each unit cell was made up of a combination of struts and free spaces.

**Figure 16:**
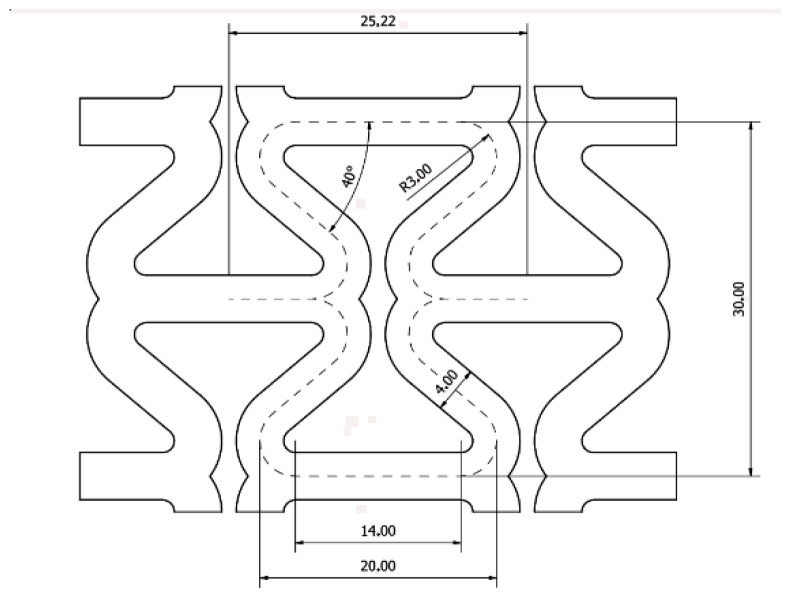
Structure consisting of two modified re-entrant cells, with the inter-strut angle of 40° marked, i.e., θ_0_ = 50°.

The study took into account the insights from the literature regarding the reduction in stress accumulating at the arched joints. The mesh element made up of re-entrant cells is shown in Figure 16.

The mesh structure produced through 3D printing is marked by a great degree of precision and repeatability on a macro scale.

The idea of replacing sharp-angled strut joints with arched ones is widely known [47,48,49,50,51,52]. This arched joint configuration has been found to allow for a higher level of elastic deformation of the metamaterial. The arched joints allow the struts to bend more easily.

Mesh structures with dimensions of 6 × 6 and unit cells with inter-strut angle values of 40, 50, 60, and 70° (i.e., θ_0_ = 50, 40, 30, and 20°) were produced by 3D printing using a FIBERFLEX 40D filament, which has good elastic properties. The structures were subjected to tensile tests using a testing machine. Two series of tests were performed; in the first one, the elastic properties of the meshes were recognised, while in the second one, the meshes were subjected to maximum tension to the point of straightening the inclined struts.

**Figure 17:**
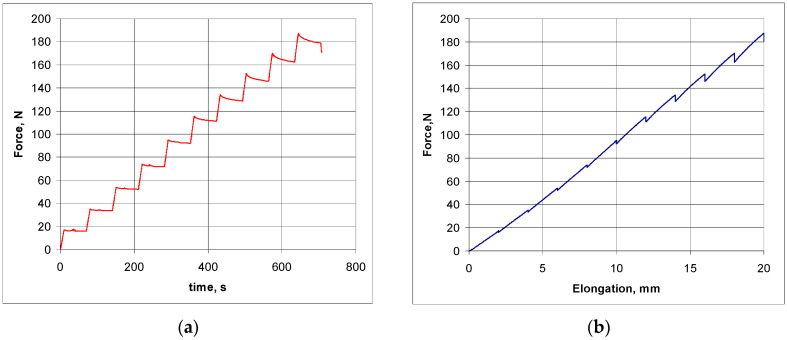
Representative results of tensile testing of a mesh structure with an initial size of 150 × 182 mm containing 6 × 6 cells using a testing machine.

The first series of tests consisted of stretching the structure at a rate of 10N/min with one-minute breaks after each step of 20 mm (Figure 17a)—to take measurements in the lateral direction (X1). The tensile test results are shown in Figure 17b.

The stress–strain curve (Figure 17b) shows a forced elongation in the vertical direction (X2). In the case of the polymer material used, there was a local decrease in elongation during the breaks with a constant tensile load. Despite this applied procedure for determining the size of the meshes in tension, a linear relationship was obtained between the tensile load and elongation in the vertical direction (X2), with an elongation of approximately 11%.

The stretched meshes showed a uniform expansion of the unit cells, but up to a certain limit. In this case, the yield strength was not approached, but an edge effect occurred, stemming from the way the meshes were fixed. This edge effect involved the ‘rippling’ of the meshes and the formation of heterogeneous areas. Once the effect appeared, the stretching was stopped.

**Figure 18:**
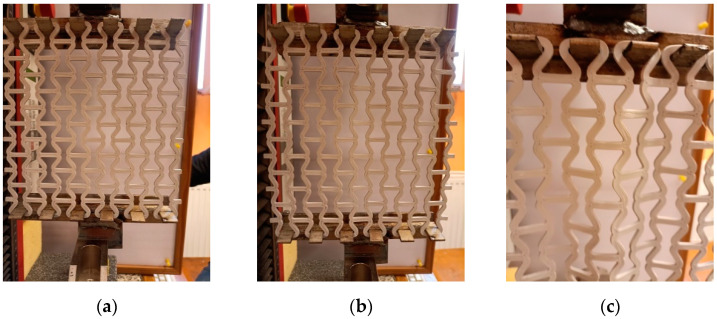
Mesh structure subject to uniaxial tensile load; initial condition (**a**); stretched mesh (**b**) and stretched mesh with ripple effect (**c**).

The ‘rippling’ effect can be seen in the following photographs—Figure 18c.

The featured photographs (Figure 18) show that the top and bottom edges of the mesh were under tension, and the uniform load in the vertical direction was balanced by the elasticity of the re-entrant cell material, with the inclined struts of the cell moving away from each other.

**Figure 19:**
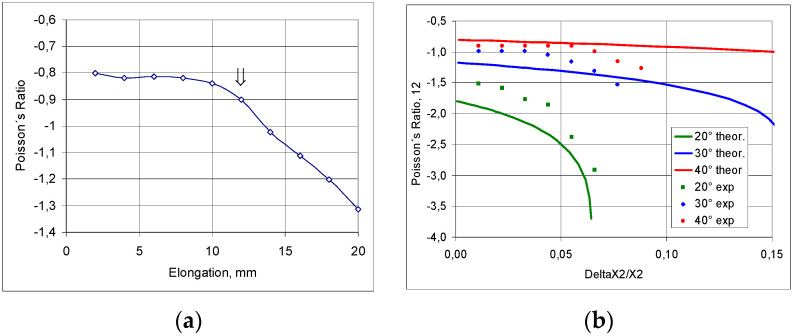
Tensile results for a grid made up of cells with an angle of 40° with the marked onset point of the ‘ripple’ effect (**a**) and the theoretical and experimental Poisson’s ratio values for meshes with different θ_0_ angle values (**b**).

On the basis of the obtained measurements, diagrams were produced showing the changes in the Poisson’s ratio for meshes with different values for the inter-strut angle (Figure 19). The Poisson’s ratio values are given for the range in which the edge effect had not yet occurred for the stretched meshes.

The relationship shown in Figure 19a shows at which point the ‘ripple’ effect arose. This unexpected deformation effect led to an apparent increase in the NPR.

Theoretically, the highest NPR values are obtained for the maximum stretching of the mesh. The lower the value of the initial angle (θ_0_), the smaller the stretching range, which yields, however, a larger NPR. Conversely, for larger values of the initial angle (θ_0_), the elongation becomes greater, with a smaller NPR.

The experimental results obtained for the elastic range (below the ‘ripple’ onset point) show that by varying the cell parameters, including the θ_0_ angle, a higher NPR is obtained.

**Figure 20:**
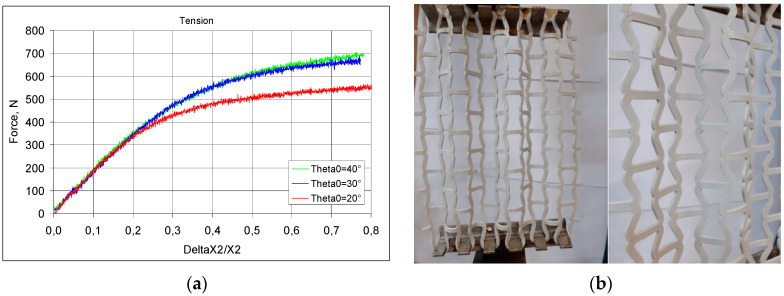
Tensile test results of meshes with elongation in relation to tensile load—(**a**), and images of meshes subjected to tension—(**b**).

In a second series of tests, the meshes were subjected to maximum tensile loads. Very high elongations in the vertical direction ΔX2/X2 were obtained in this case—Figure 20a.

In tension, there is an observed increase in the gap between the inclined struts (Figure 20b) and a lateral expansion of the structure.

The obtained results indicate that irrespective of the geometry of the modified re-entrant cell, the range of elongation is linear. Above the value of around 0.2, the relative change in dimensions ΔX2/X2 became curvilinear, with the values for tensile load becoming smaller the smaller the initial angle θ_0_. The final elongation of the meshes obtained depended not only on the aforementioned θ_0_ angle, but mainly on the material of the cells. This indicates that both the material and the structural design affect the behaviour of the mesh in tension.

By comparing the experimental results with the theoretical calculations, it is possible to determine the accuracy of the predictions. Theoretical calculations show that, for the meshes under consideration, full straightening of the inclined struts of the re-entrant cells takes place at specific values of ΔX2/X2, which is 86.4% for θ_0_ = 40°, 56.4% for θ_0_ = 30°, and 32.3% for θ_0_ = 20°. Such points are absent, however, from the experimentally obtained curves. The accompanying photographs (Figure 20b) show the processes involved in stretching the meshes: the straightening of the inclined struts and the aforementioned rippling of the mesh. The modified re-entrant structures also exhibit expansion in the direction perpendicular to the tension, thus achieving auxetic behaviour.

Another characteristic feature of the studied meshes is the fact that after the tensile tests and with force no longer applied, both their vertical and horizontal dimensions were larger compared to their initial dimensions, i.e., there was a permanent deformation in both orthogonal directions, which was around 20% in the direction of the tension and around 6% in the perpendicular direction.

It can be added that the stretched meshes change their shape as predicted and then undergo specific uncontrolled deformations—dependent on both the measurement conditions and the material properties of the re-entrant cells.

### Discussion of Mesh Structures

Mesh-like metamaterial structures made of bow-tie elements are particularly popular due to their potential applications. Their auxetic behaviour is studied through tensile tests. Numerous publications have investigated the simultaneous elongation in orthogonal directions both theoretically (through simulation) and experimentally—with concrete physical models [27,48,53,54,55]. For the most part, these studies have mainly focused on the elastic properties and small deformations of metamaterials. They were done, therefore, within the linear elastic range.

It is typical for such structures to exhibit relatively low elongation in tension, and any attempts to increase their elongation cause damage to the structure each time.

Numerous studies show that by increasing tensile loads, various types of defects are caused, such as constrictions [56], tears [48,55] and larger breaks of continuity [54].

The ‘ripples’ found in the studies presented here are a type of local defect—connected with increased deformation. The degree to which these defects are present depends, on the one hand, on the elastic properties of the cell material as well as on the applied cell modification. The arched strut joints contribute to a greater degree of stretch in the mesh structures before they are damaged. This is also reflected in higher NPR values. It was found that a structure without strut joints of this type exhibited a lower NPR, and for structures with an angle of θ_0_ = 40°, this difference was around 0.2. A similar trend was already observed in an earlier study [54].

The present study found that the proposed flexibility-oriented approach is largely dependent on the way the mesh is fixed (boundary conditions).

In tension, an increase in the spacing of the inclined struts (Figure 18b) and horizontal expansion of the mesh was observed. As a result, each unit cell expanded in all directions. The main mechanism of structural deformation is the bending of the arched strut joints. However, with the elongation exceeding 15/185 (for θ_0_ = 40°), a ripple effect occurred and measuring the dimensions of the structure in the horizontal direction (X1) became problematic. Elongation up to approximately 20/185 remained reversible, although certain inhomogeneities were created (Figure 18c). This deformation occurred mainly in the central rows of the mesh. Similar effects have already been observed in other studies [10,54].

It seems, however, that the ripple effect and the appearance of uneven gaps are due to the method of fixing/suspending the mesh. This also indicates that at higher strain levels, edge effects become more prominent.

In conclusion, the influence of different morphological and geometrical parameters on the overall strain of meshes made of re-entrant cells was demonstrated experimentally. This approach could prove to be an effective tool in the design of programmable mechanical metamaterial components of adaptable shapes.

The proposed design method can find a wide range of applications, e.g., in soft robotics, the fashion industry, and healthcare.

## 6. Conclusions

To ensure an adequate treatment of auxetic structures formed from re-entrant cells, a geometrical analysis of such structures was first carried out. The analytical relationships obtained provide a general framework for analysing the dimensional changes of structures—their displacement as a result of external mechanical forces. Such structures constitute an ordered arrangement of interconnected struts and free spaces. As a result of external loads, the structures can undergo deformations, which can be in the form of controlled dimensional changes—if they occur within the elastic range of the strut material. Such deformation of the struts and change in the dimensions of the structure can occur if there is a change in the Δθ angle. If Δθ = 0°, the structure is locked and does not undergo dimensional changes, thus not exhibiting auxetic properties nor achieving NPR. In the particular case of Δθ→0, but for Δθ ≠ 0, it is possible to find the limit value of the Poisson’s ratio for a re-entrant cell structure. A number of publications cite this value as one of the characteristics of the structure. It seems that such an approach, although useful in simulations, is not applicable in physical reality.

It has been shown experimentally that the dimensional change in structures does not take place by hinging the struts but only by bending the strut joints, which can occur for both the elastic range and the plastic range of the strut material. The struts can also expand or contract under very high loads—as happens, for example, in the case of their abrupt failure.

The geometry of the re-entrant cells, expressed by the ratio of strut lengths h/l with the condition h/l < 2, has a decisive influence on the potential for changes in the dimensions of the structure. In this case, the value of the initial angle θ_0_ must be less than the value of the angle θ_limit_ = arcsin(h/2l). In the compression of such structures (though not to the point of failure), the value of the angle θ = θ_0_ + Δθ satisfies the condition θ < θ_limit_.

The above restrictions do not apply for re-entrant cells for which h/l ≥ 2 is true. Although, in general, the change in the Δθ angle associated with tension can go from an initial value of θ_0_ to 0°. For compression, on the other hand, the change in the Δθ of the angle can go from the initial value θ_0_ to θ_limit_—for h/l < 2—and from θ_0_ to 90° for h/l ≥ 2. In practice, re-entrant cells cannot reach the extreme values (i.e., 0° or 90°) of the θ angle because that would make the structure effectively lose its re-entrant character without the possibility of returning to the original state.

Structures made of re-entrant cells exhibit auxetic properties when subjected to strain, and they can achieve especially large NPR values, though only for particularly adjusted values of the parameters h/l θ_0_ and Δθ, e.g., for stretching structures with parameters h/l < 1 (long narrow cells) and at the same time for small initial θ_0_ angles and small Δθ angle changes. In tension, a maximum NPR is obtained for a maximally stretched structure (θ = θ_0_ − Δθ→0°).

In compression, a high NPR is obtained for h/l < 1 and for very small angle changes (Δθ→0°).

As a reminder, high NPR values mean that in one direction, there is a positive change in the structure’s linear dimensions as a result of the applied force, while in the perpendicular direction, the change is also positive, albeit very small. In practice, this means that for a large NPR, the structure expands in one direction, while orthogonally, it remains almost unchanged.

To illustrate the general theoretical relationships discussed, experimental models of structures made of re-entrant cells have been presented. For compression tests, a symmetrical structure of four re-entrant cells was assembled, while for tensile tests, meshes of 6 × 6 cells were 3D printed. Both compression and tensile tests (for the elastic ranges of the unit cell material) yielded results that were in good agreement with the theoretical ones.

The chosen polymer materials (PVC for compression tests and FIBERFLEX 40D for tensile tests) exhibited satisfactory properties required for the structures tested. The symmetrical PVC structure proved its adequacy for compression tests. During the assembly and glueing of the struts, it was noted that very small inaccuracies and poor adhesion of some of the horizontal struts led to cell expansion rather than contraction when compressed.

When it comes to the tensile tests, the 3D printed mesh structures also proved to be well suited for the process. It was noted that the way of fixing the meshes significantly affected the tension and expansion process in the direction perpendicular to the tensile load. In the case of the fixing elements used for stretching the meshes, a ‘ripple’ effect of the structure occurred at some stage of the stretching, which was an edge effect of the experiment.

In summary, the present work has thus re-examined the auxetic structures made up of re-entrant cells, demonstrating the possible ways of their functioning. This can be viewed as bridging the gap between the virtual reality in which such structures are typically conceived and the physical reality of their actual implementation.

## Figures and Tables

**Table 1:** The relationships for the change in the horizontal X1 and vertical X2 linear dimensions of structures made of re-entrant cells.

$\Delta X1=2(n-1)l[-2l\sin(\theta_{0}\pm \Delta \theta )+2l\sin\theta_{0}]$ $\frac{\Delta X1}{X1}=\frac{\left(n-1)2l(\sin\theta_{0}-\sin(\theta_{0}\pm \Delta \theta )\right)}{nh+(n-1)[h-2l\sin\theta_{0}]}$ $\frac{\Delta X1}{X1}=\frac{\sin\theta_{0}-\sin(\theta_{0}\pm \Delta \theta )}{\frac{2n-1}{2(n-1)}\frac{h}{l}-\sin\theta_{0}}$	$\Delta X2=2ml(\cos(\theta_{0}\pm \Delta \theta )-\cos\theta_{0})$ $\frac{\Delta X2}{X2}=\frac{2ml(\cos(\theta_{0}\pm \Delta \theta )-\cos\theta_{0})}{2ml\cos\theta_{0}}$ $\frac{\Delta X2}{X2}=\frac{\cos(\theta_{0}\pm \Delta \theta )-\cos\theta_{0}}{\cos\theta_{0}}$
Whereas, with the increase of n, the fraction	
$\frac{2n-1}{2(n-1)}$	
tends towards the value of 1, for tension it occurs that θ < 0, and for compression it occurs that Δθ > 0, where n and m are the number of cells in the structure.	

## Data Availability

All data are contained within the article.
